# *In-situ* Grown SnS_2_ Nanosheets on rGO as an Advanced Anode Material for Lithium and Sodium Ion Batteries

**DOI:** 10.3389/fchem.2018.00629

**Published:** 2018-12-18

**Authors:** Hezhang Chen, Bao Zhang, Jiafeng Zhang, Wanjing Yu, Junchao Zheng, Zhiying Ding, Hui Li, Lei Ming, D. A. Mifounde Bengono, Shunan Chen, Hui Tong

**Affiliations:** ^1^School of Metallurgy and Environment, Central South University, Changsha, China; ^2^School of Chemistry and Chemical Engineering, Central South University, Changsha, China

**Keywords:** SnS_2_, reduced graphene oxide, thin nanosheets, anode material, lithium ion batteries, sodium ion batteries

## Abstract

SnS_2_ nanosheets/reduced graphene oxide (rGO) composite was prepared by reflux condensation and hydrothermal methods. In this composite, SnS_2_ nanosheets *in-situ* grew on the surface of rGO nanosheets. The SnS_2_/rGO composite as anode material was investigated both in lithium ion battery (LIB) and sodium ion battery (SIB) systems. The capacity of SnS_2_/rGO electrode in LIB achieved 514 mAh g^−1^ at 1.2 A g^−1^ after 300 cycles. Moreover, the SnS_2_/rGO electrode in SIB delivered a discharge capacity of 645 mAh g^−1^ at 0.05 A g^−1^; after 100 cycles at 0.25 A g^−1^, the capacity retention still keep 81.2% relative to the capacity of the 6th cycle. Due to the introduction of rGO in the composite, the charge-transfer resistance became much smaller. Compared with SnS_2_/C electrode, SnS_2_/rGO electrode had higher discharge capacity and much better cycling performance.

## Introduction

Lithium ion batteries (LIBs) are being widely used in the electric vehicles and energy storage fields (Wu et al., 2017; Chen et al., 2018b,c; Cui et al., 2018; Zhang et al., 2018; Zheng et al., 2018). However, the commercial graphite anode is far to meet the requirements of the high-performance LIBs due to its low theoretical capacity (Ryu et al., 2016; Li et al., 2017b; Yan et al., 2017; Wang et al., 2018). Furthermore, lithium resource is limited in nature. It is necessary to develop low cost and high storage performance sodium ion batteries (SIBs) to satisfy the energy demand (Li et al., 2017a; Zhu et al., 2017; Chen et al., 2018a). Sodium is rich on the earth, and possesses similar physical and chemical properties as lithium (Qu et al., 2014; Jiang et al., 2015; Wang et al., 2015b; Zhang et al., 2015a, 2017a; Fang et al., 2016; Xu et al., 2016c; Leng et al., 2017). However, the commercial graphite material is not suitable for SIBs (Qu et al., 2014; Fang et al., 2016; Xu et al., 2016c; Zhang et al., 2016a). Therefore, it is urgent to develop new anode materials with low cost and good electrochemical properties for SIBs.

Currently, many researches have been carried out in transition metal oxides (Zhang et al., 2016b), metals (Lin et al., 2013) and carbonaceous materials (Xu et al., 2016a). These anode materials were used in both LIBs and SIBs, but suffer from the disadvantages of poor sodium storage, poor cycling property, etc. The layered structure materials, such as SnS_2_, have been considered as promising anode materials for SIBs (Chao et al., 2016), due to high theoretical capacity, electrochemical stability, low cost and environmental friendliness. SnS_2_ has a layered structure with a large interlayer distance which can promote the intercalation and deintercalation of lithium and sodium ions. SnS_2_/C prepared by using polyacrylonitrile as carbon source delivered the capacity of 570 mAh g^−1^ after 100 cycles at discharge current density of 0.05 A g^−1^ (Wang et al., 2015b). However, SnS_2_ electrode still suffers the poor electrochemical performance, such as large cycling capacity loss for its low electronic conductivity. To overcome these problems, graphene was introduced into the anode materials. The graphene can improve the electric conductivity and decrease the volume change during the intercalation and deintercalation process of the lithium and sodium ions. So, the electrochemical performance of the anode material could be improved by reduced graphene oxide (rGO) introduction. Du et al. reported that, after 50 cycles, Co_3_S_4_-PNS/graphene sheet electrode still show the discharge capacity of 329 mAh g^−1^ at 0.5 A g^−1^ (Du et al., 2015). SnSe_2_ nanoplate/graphene composite was synthesized by hydrothermal method and showed a better storage performance than that of SnSe_2_ nanoplates without graphene (Choi et al., 2011). Zhang et al. reported that FeSe_2_/sulfur-doped rGO sheets displayed the discharge capacities of 383.3 and 277.5 mAh g^−1^ at high current densities of 2.0 and 5.0 A g^−1^ (Zhang et al., 2016a). Nanosheet materials could be also prepared by solution combustion synthesis method (Ramkumar and Minakshi, 2015; Ramkumar and Sundaram, 2016).

In this work, a novel method was developed to synthesize SnS_2_/rGO composite anode material for LIBs and SIBs. In this composite, SnS_2_ grew on the surface of rGO nanosheets, which possessed good electronic conductivity. So, the SnS_2_/rGO electrode exhibited outstanding lithium and sodium storage properties and cycling performance. The preparation mechanism, as well as the physical and electrochemical properties of SnS_2_/rGO composite was carefully discussed.

## Experimental

Graphene oxide (GO) was dispersed in deionized water, with the concentration of 1.5 mg mL^−1^. 20 mL ethylene glycol and 30 mL GO solution were introduced into a round-bottom flask. The mixed liquid was ultrasonically treated for 0.5 h. 0.5438 g SnCl_4_·5H_2_O dissolved in 10 mL EG was then added into the mixed liquid. After magnetically stirred for 0.5 h, the suspension was heated to 120°C and treated by reflux condensation method for 2 h, and then cooled down to the room temperature. 0.6014 g thioacetamide and the suspension were added to a 100 mL Teflon-lined stainless steel autoclave and then magnetically stirred for 20 min. Then, the autoclave was transferred into an oven and kept at 160°C for 12 h. After cooling down naturally to room temperature, the precipitate was centrifuged, and washed by deionized water and absolute alcohol for several times, and then dried at a 80°C vacuum oven for a whole night. The mixture was sintered at 500°C for 4 h under Ar atmosphere. Finally, SnS_2_/rGO composite with 15 wt% rGO was obtained. The SnS_2_/rGO composites with 10 and 20 wt% rGO were also prepared through the same way by adding different contents of GO. For comparison, the SnS_2_/C composite was synthesized by the same method without using GO, replaced by 1 g glucose as the carbon source added into the suspension together with thioacetamide.

The crystalline phase of SnS_2_/rGO composite was analyzed by X-ray diffraction (XRD, Rigaku D/Max 200PC, Japan) using Cu Kα radiation. The scanning rate was 5° per minute, and the range of scanning diffraction angle (2θ) was from 10° to 80°. The Raman spectra were conducted by Raman spectroscopy (Lab RAM Aramis, Jobin Yvon, France). The oxidation states of Sn, S, and C elements in the samples were studied by X-ray photoelectron spectroscopy (XPS, PHI5700, USA). The morphologies of the samples were observed by scanning electron microscopy (SEM, FEI, Nova NanoSEM-230, USA), and high resolution transmission electron microscopy (TEM, FEI, Tecnai G2 F20 S-Twin, USA), working at 200 kV. The element contents of the samples were studied by energy dispersive X-ray spectroscopy (EDS). The carbon and sulfur analyzer (CS744, Leco, USA) was applied to quantify the amount of carbon and sulfur in the composite.

The SnS_2_/rGO anode material was evaluated using 2025-type coin cells prepared in a pure argon-filled glove box. The product slurry was prepared by mixing SnS_2_/rGO powder, carbon black and polyvinylidene fluoride by 8:1:1 in weight, and dispersed in N-methyl pyrrolidinone. The result slurry was pasted onto a Cu foil. After dried, the foil with SnS_2_/rGO material was cut to a 14 mm diameter disk. The electrolyte for SIBs was 1 M solution of NaClO_4_ dissolved in ethylene carbon (EC)/dimethyl carbonate (DMC) (weight ratio 1:1) and 5 wt% fluoroethylene carbonate. The electrolyte for LIBs was 1 M solution of LiPF_6_ in EC-DMC (weight ratio 1:1). The counter electrode for SIBs was a sodium foil and a Whatman GF/D as the separator. And lithium foil counter electrode and Celgard 2500 separator were used in LIBs. Electrochemical tests were conducted on LAND battery cycler by using an automatic galvanostatic charge-discharge unit (LAND CT2001A, China), with the potentials of 0.01–2.50 V vs. Na/Na^+^ electrode and 0.01-1.80 V vs. Li/Li^+^ electrode. The cyclic voltammetry (CV) and electrochemical impedance spectroscopy (EIS) measurements were recorded by an electrochemical workstation (CHI660D, CH Instruments, USA). The EIS spectra were recorded by applying an AC voltage of 5 mV amplitude in the frequency of 10^−2^−10^5^ Hz. The scanning rate of CV was 0.1 mV s^−1^.

## Results and Discussion

**Figure 1:**
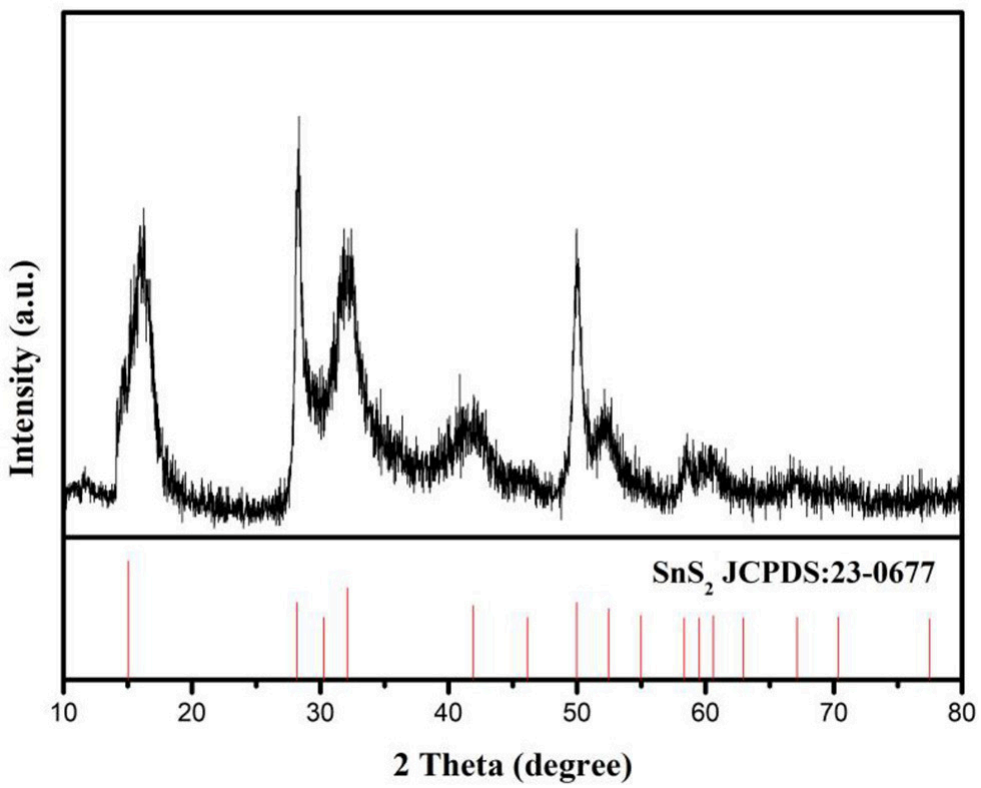
XRD pattern of SnS_2_/rGO composite.

SnS_2_/rGO composite was prepared by reflux condensation and hydrothermal methods. The XRD pattern of SnS_2_/rGO composite was showed in Figure 1. The pattern shows that all the peaks are indexed to SnS_2_ (JCPDS#23-0677) with 2T-type layered structure. There were no obvious impurity peaks in the XRD pattern, especially at 10.9° which is the location of characteristic peak of GO. It means that there is no impurity in SnS_2_ sample and GO was reduced to rGO through heat treatment.

**Figure 2:**
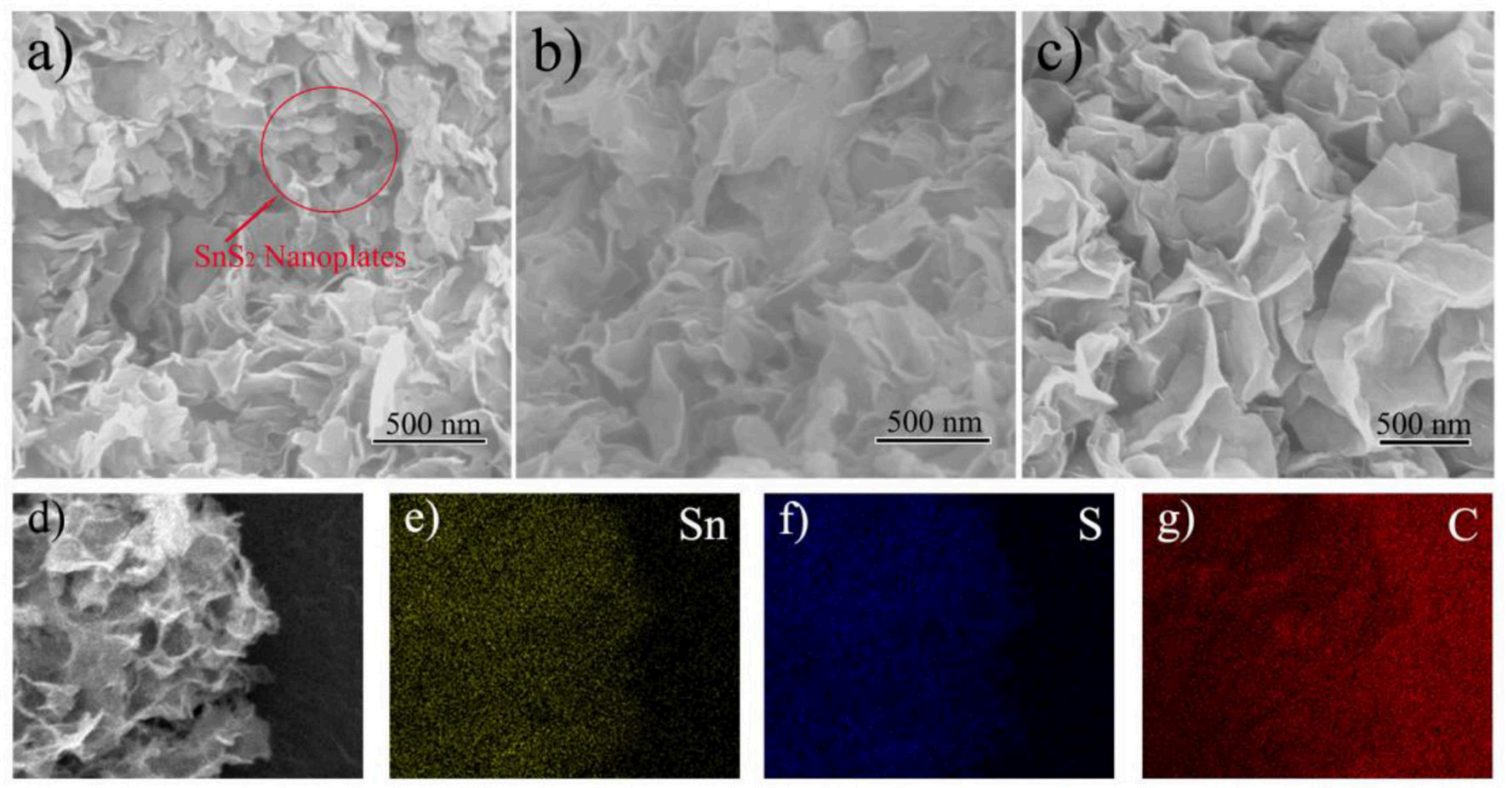
SEM images of SnS_2_/rGO composites with different contents of rGO: **(A)** 10 wt%, **(B)** 15 wt%, **(C)** 20 wt%, and **(D)** 15 wt%; the elemental mappings of **(E)** Sn, **(F)** S, and **(G)** C.

The SEM images of SnS_2_/rGO composites with different contents of rGO were shown in Figure 2. The designed contents of rGO in the composites were 10 wt% (Figure 2A), 15 wt% (Figure 2B) and 20 wt% (Figure 2C). In Figure 2A, it can be observed that SnS_2_ not only grew on rGO nanosheets but also self-assembled as nanoplates. When the rGO content increased to 15 wt%, there were no SnS_2_ nanoplates observed in Figure 2B and the thickness of SnS_2_/rGO sheets became thicker than that of rGO sheets (not shown). In Figure 2C, the morphology of the sample seems no obvious difference with that of the sample in Figure 2B. The rGO in the samples can be regarded as a template for the growth of SnS_2_. SnS_2_ self-assembled as nanoplates when the content of rGO was not enough. And the SnS_2_ nanoplates disappeared as the rGO content increased in the composite. The rGO content in the composite (Figure 2C) is excessive, which reduce the volume energy density and raise product cost. So, the optimum content of rGO was 15 wt% among the three samples. The measured content of S element in the composite (Figure 2B) was 29.53 wt%. So, the content of rGO in the composite was calculated as 15.8 wt%, which is close to the designed value of 15 wt%. The properties of SnS_2_/rGO (15 wt%) composite were further investigated.

**Figure 3:**
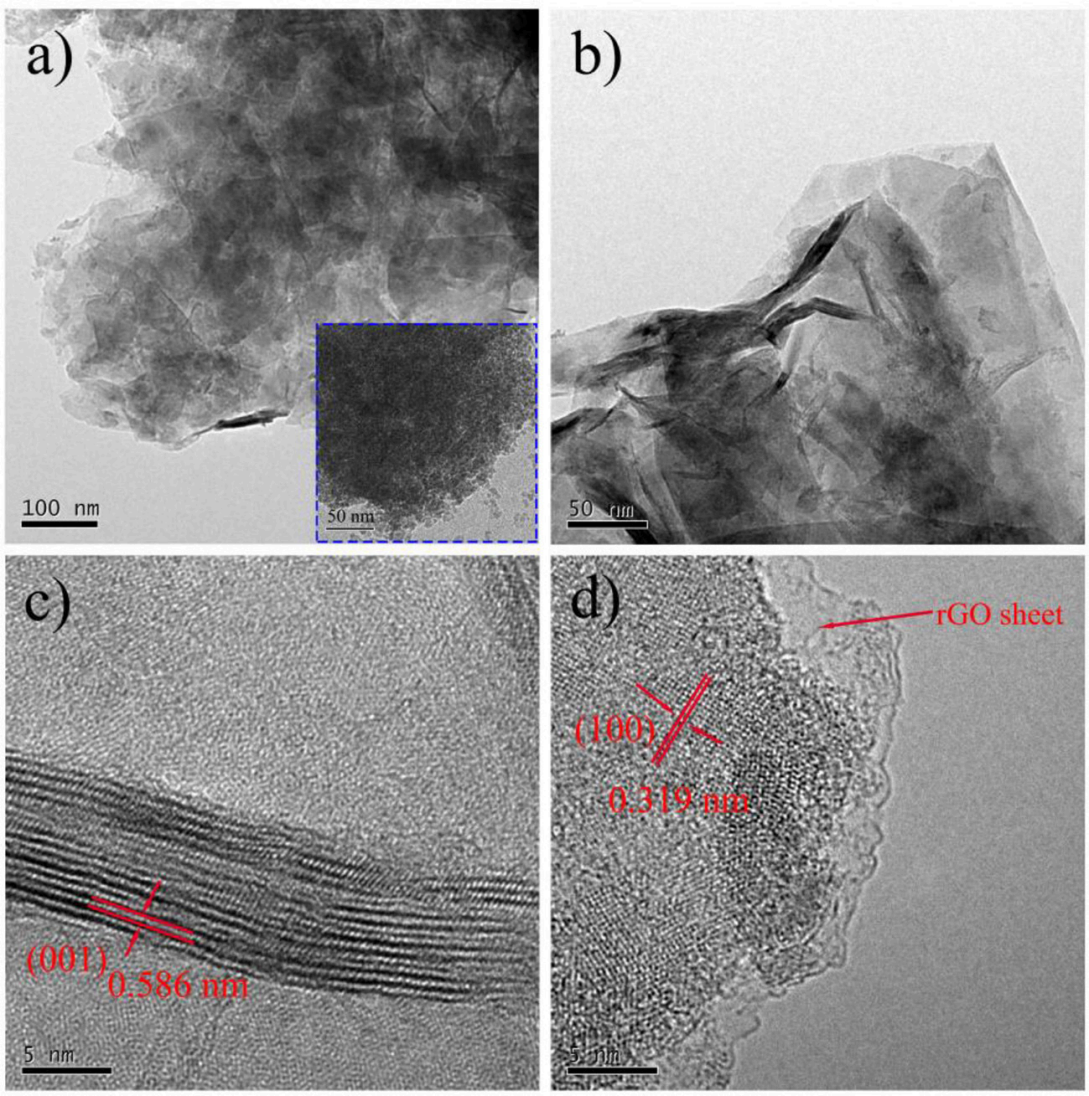
**(A,B)** TEM images and **(C,D)** HRTEM images of SnS_2_/rGO composite; the insert in **(A)** is the TEM image of SnO_2_/GO composite.

The distributions of Sn, S, and C elements of SnS_2_/rGO composite were studied by EDS. The EDS mappings further show that SnS_2_ was distributed homogeneously on rGO nanosheets. The microstructure of SnS_2_/rGO nanosheets was conducted by TEM, as shown in Figure 3. In Figure 3A, the TEM image shows that SnS_2_ nanosheets were coated by the plicate graphene nanosheets. The inset figure in Figure 3A shows that SnO_2_ nanoparticles grew on the GO nanosheets before the hydrothermal synthesis of SnS_2_/rGO. The SnO_2_ nanoparticles were about 5–10 nm and coated on the surface of GO nanosheets tightly. Thus, SnS_2_ nanosheets could *in-situ* grow on the surface of GO nanosheets. The side view of the SnS_2_ nanosheets is shown in Figure 3B, and it is seen that the thickness of the nanosheets is about 10 nm. The parallel fringe spacing in Figure 3C was 0.586 nm and the lattice distance in Figure 3D was 0.319 nm, corresponding to (001) plane and (100) plane of SnS_2_ with 2T-type layered structure, respectively. There is no obvious crack or collapse in SEM and TEM images, which is beneficial for good cycle stability of the composite.

**Figure 4:**
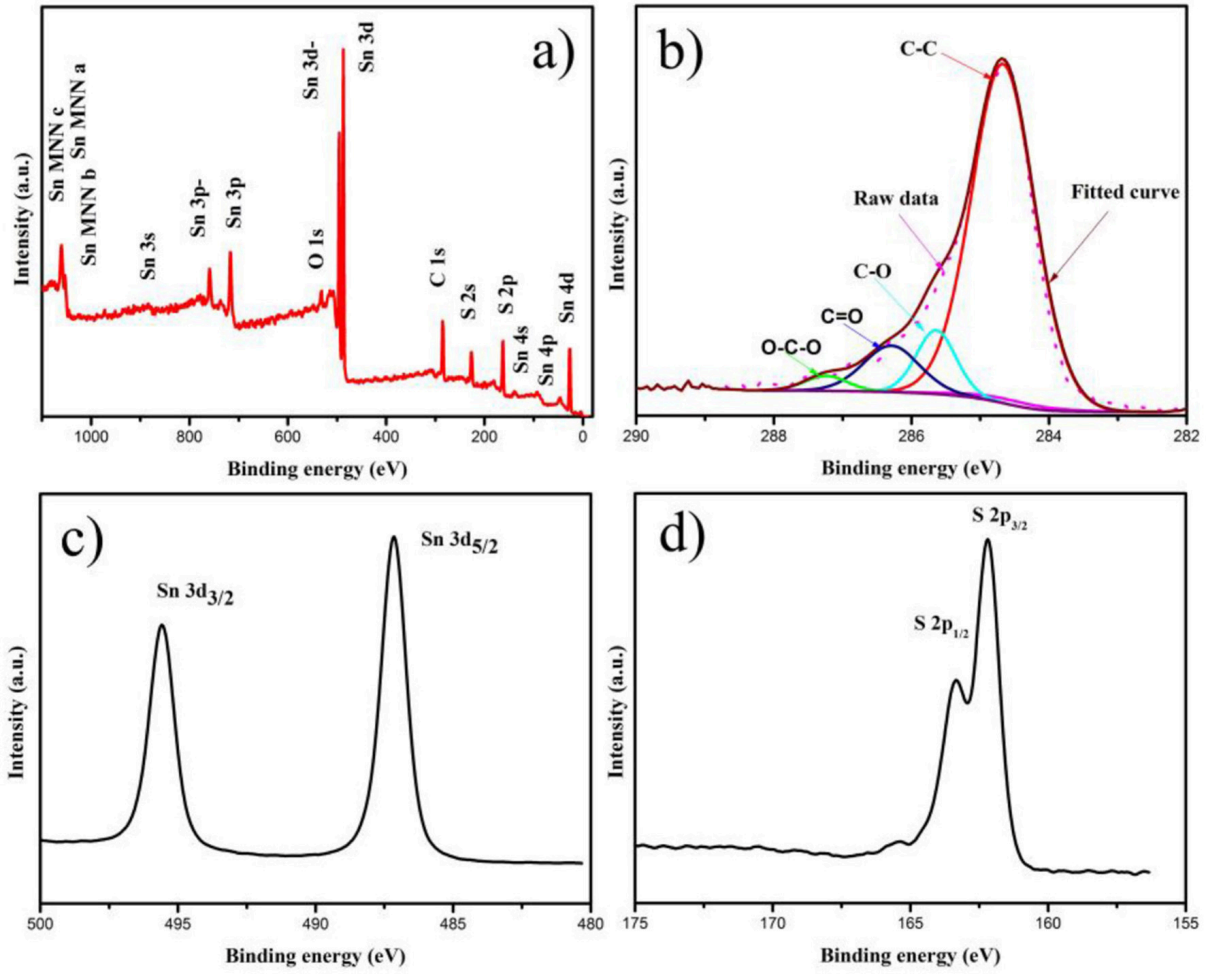
**(A)** XPS spectrum of SnS_2_/rGO composite; the high-resolution XPS spectra of **(B)** C 1 s, **(C)** Sn 3d, and **(D)** S 2p.

The XPS analysis was applied to investigate the electronic states of Sn, S, and C elements in SnS_2_/rGO composite, as shown in Figure 4. The Sn 3d, C 1s and S 2p peaks can be observed clearly in Figure 4A. In Figure 4B, the C 1s peak was resolved into four parts. The peaks centered at 284.6, 285.6, 286.3, and 287.3 eV correspond to C-C, C-O, C = O and O-C = O type bonds, respectively (Qu et al., 2014; Zhang et al., 2014; Fang et al., 2016). In Figure 4C, the high-resolution Sn 3d spectrum exhibited two signals at 487.2 and 495.6 eV for Sn 3d3/2 and Sn 3d5/2, respectively, corresponding to Sn^4+^. In Figure 4D, the presence of SnS_2_ can be confirmed by S 2p peak at 163.4 and 162.3 eV (Zhang et al., 2015b).

**Figure 5:**
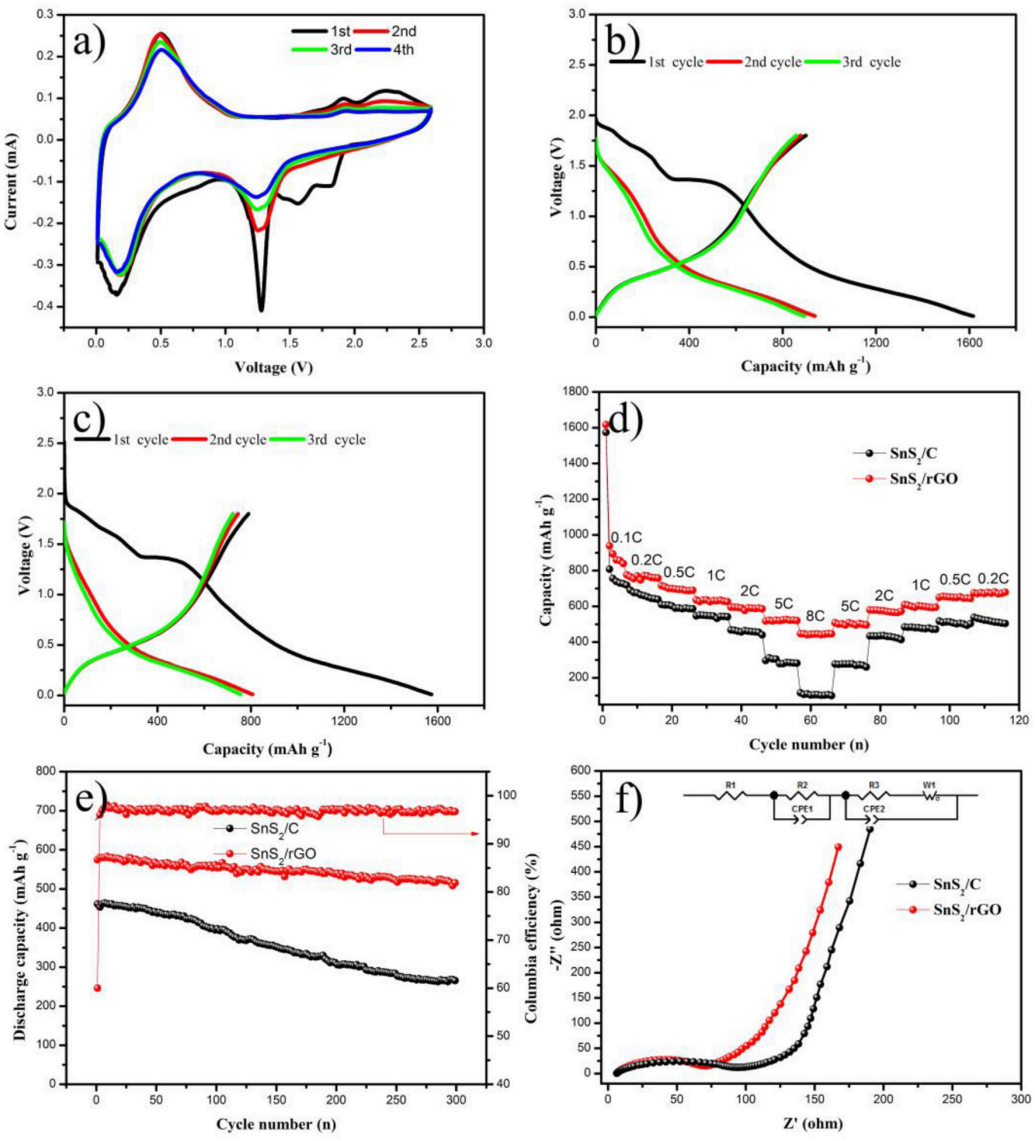
Electrochemical performances of SnS_2_/rGO and SnS_2_/C electrodes in LIBs: **(A)** CV curves of SnS_2_/rGO electrode; charge and discharge curves of **(B)** SnS_2_/rGO and **(C)** SnS_2_/C electrodes; **(D)** rate and **(E)** cycle performances of the electrodes; **(F)** Nyquist plots of the electrodes; the inset is the equivalent circuit for EIS results fitting.

The electrochemical performances of the samples were investigated by coin cells. Figure 5 shows the lithium storage, rate and cycling properties. The CV test of SnS_2_/rGO electrode was conducted in the voltage of 0.01–2.6 V. The alloying and dealloying reactions happened between Li and SnS_2_, as described in Equations (1) and (2) (Zhai et al., 2011). In the first cycle of cathodic scans, Li_x_SnS_2_ formed at 1.8 V by the lithium intercalation into SnS_2_ layers. Two broader peaks were present at about 1.3 and 0.12 V. The peak at about 1.3 V corresponds to the decomposition of the SnS_2_ into metal Sn and Li_2_S. The peak at about 0.12 V is ascribed to the formation of Li_x_Sn by lithium and metal Sn (Zhai et al., 2011; Zhang et al., 2014). The peaks in 1.8-2.6 V correspond to the formation of SnS_2_. In the anodic scans, the peaks appeared at around 0.5 V are mainly related to the dealloying of Li_x_Sn. Figures 5B,C show the typical charge and discharge profiles of SnS_2_/rGO and SnS_2_/C electrodes at 600 mA g^−1^, respectively. The charge and discharge voltage was between 0.01 and 1.8 V. The SnS_2_/C electrode exhibited initial discharge and charge capacity 1574 and 790.1 mAh g^−1^, with a coulombic efficiency of 50.2%. However, SnS_2_/rGO electrode delivered higher discharge and charge capacity of 1616.6 and 899.6 mAh g^−1^, with higher initial coulombic efficiency of 55.6% compared with that of SnS_2_/C electrode (50.2%).

(1) $$\begin{array}{l} \text{Sn}{\text{S}}_{2}+4\text{ Li}\to \text{Sn}+2\text{L}{\text{i}}_{2}\text{S} \end{array}$$

(2) $$\begin{array}{l} \text{Sn}+4.4\text{ Li}\to \text{L}{\text{i}}_{4.4}\text{Sn} \end{array}$$

Rate capability is highly crucial for anode materials in LIBs, and the rate performances of the samples are shown in Figure 5D. SnS_2_/rGO electrode exhibited outstanding rate performance compared with that of SnS_2_/C electrode. SnS_2_/rGO electrode showed high discharge capacities of 776, 715, 635.6, 595.2, 517.5 and 447.1 mAh g^−1^ at 0.2, 0.5, 1, 2, 5 and 8 C, respectively. After cycling at different current densities, SnS_2_/rGO electrode delivered 675 mAh g^−1^ at 0.2 C. However, SnS_2_/C composite as a contrastive electrode showed worse rate performance, achieving the discharge capacities of 718.2, 609.7, 546.7, 467.7, 297 and 116 mAh g^−1^ at 0.2, 0.5, 1, 2, 5 and 8 C, respectively. Figure 5E shows the cycling properties of SnS_2_/C and SnS_2_/rGO electrodes at 1.2 A g^−1^. Obviously, for SnS_2_/rGO electrode, a higher discharge capacity of 514 mAh g^−1^ can be obtained after 300 cycles. Compared with SnS_2_/rGO electrode, the SnS_2_/C electrode showed a worse cycle performance. The cycle performance of SnS_2_/rGO electrode in this work and other literatures were summarized in Table 1. It is found that SnS_2_/rGO electrode in this work exhibited an excellent cycle performance.

**Table 1:** Rate performances of SnS_2_/rGO electrodes in this work and the other literatures.

**Samples**	**Voltage window (V)**	**Discharge capacity (mAh g^**−1**^)**	**Current density (mA g^**−1**^)**	**Cycle number**
SnS_2_ NS@MWCNTs (Zhai et al., 2011)	0.01–1.15	400	100	50
G-SnS_2_-S (Luo et al., 2012)	0.005–1.3	650	100	30
SnS_2_/MWCNTs (Sun et al., 2014)	0.005–1.15	528	100	50
SnS_2_ Nanoplates (Seo et al., 2008)	0.001–1.1	583	323	30
FL-SnS_2_/G (Chang et al., 2012)	0.01–1.5	920	100	50
Carbon-coated SnS_2_ (Kim et al., 2009)	0–1.2	668	50	50
CC-VN@SnS_2_ (Balogun et al., 2015)	0.01–3	791	650	100
SnS_2_@PANI (Wang et al., 2015a)	0.01–3	730.8	100	80
SnS_2_@GF (Ren et al., 2016)	0.01–2.5	818.4	1,000	500
SnS_2_ nanoflower (Guan et al., 2016)	0.01–1.2	431.8	100	50
TSG (Zhang et al., 2014)	0.01–3	1,005	100	200
CPN@SnS_2_ (Chen et al., 2017)	0.01–2	699.2	60	100
SnS_2_ nanoplates (Wang et al., 2013a)	0.005–1.2	543	100	50
Ce-SnS_2_ (Wang et al., 2013b)	0.01–2.5	450.7	90	50
This work	0.01–1.8	514	1,200	300

To further understand the reason for the improvement of electrochemical properties of SnS_2_/rGO electrode, the EIS of which was measured. Figure 5F displays the Nyquist plots of SnS_2_/rGO and SnS_2_/C electrodes. The curves exhibited similar shapes, including a semicircle and a straight line in the high frequency region and the low frequency region, respectively. The inset figure of Figure 5F is an equivalent circuit model, which is the fitting result of the Nyquist plots. R_1_ is the ohmic resistance of the electrolyte and electrode; R_ct_ is the charge transfer resistance, the value of which is the sum of R_2_ and R_3_; Z_ω_ is the Warburg impedance; CPE represents the double layer capacitance and passivation film capacitance (Zhang et al., 2017b; Tong et al., 2018). R_1_ of SnS_2_/rGO and SnS_2_/C electrodes was 6.4 and 6.2 Ω, respectively; R_ct_ of SnS_2_/rGO was 79.7 Ω, which is much smaller than that of the SnS_2_/C electrode (105.2 Ω). The decrease of R_ct_ in SnS_2_/rGO electrode implies that the charge transfer process was successfully facilitated by rGO nanosheet introduction (Xu et al., 2016b).

**Figure 6:**
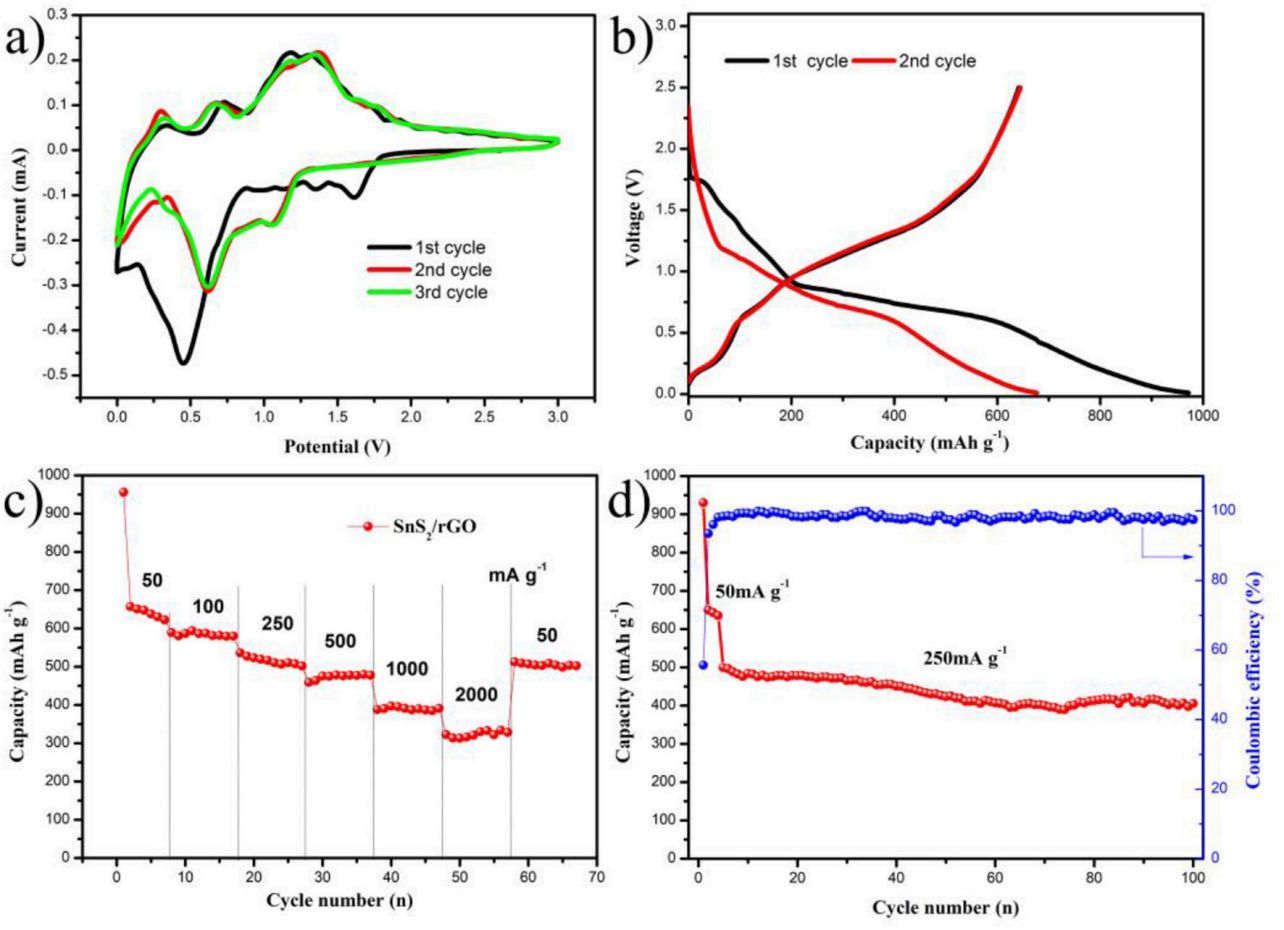
Electrochemical performance of SnS_2_/rGO electrode in SIBs: **(A)** CV curves of the electrode; **(B)** charge and discharge curves of the electrode; **(C)** rate and **(D)** cycle performances of the electrode.

The sodium energy storage performance was also investigated and shown in Figure 6. The CV for the first three cycles of SnS_2_/rGO electrode was shown in Figure 6A, which was tested between 0.01 and 3.00 V. The electrochemical reactions described in Equations (3) and (4) (Zhang et al., 2015c). The peak at about 1.6 V corresponds to the intercalation of sodium into SnS_2_ layers and the formation of Na_x_SnS_2_ during the first cathodic scan (Qu et al., 2014; Wang et al., 2015b; Chao et al., 2016). The peak at about 0.45 V is ascribed to the conversion and alloying reactions. And the peak was shifted to about 0.6 V in the second and third cycles. It is due to the formation of solid electrolyte interface (SEI) film. In the anodic scans, there were three peaks at about 0.3, 0.75, and 1.3 V, which correspond to the formation of metal Sn, Na_x_SnS_2_, and SnS_2_, respectively. The second and the third cycles were almost the same, which suggests that SnS_2_/rGO electrode possesses a good reversibility during the sodiation and desodiation process.

(3) $$\begin{array}{l} \text{Sn}{\text{S}}_{2}+4\text{ Na}\to \text{Sn}+2\text{N}{\text{a}}_{2}\text{S} \end{array}$$

(4) $$\begin{array}{l} \text{Sn}+3.75\text{ Na}\to \text{N}{\text{a}}_{3.75}\text{Sn}\left(\text{N}{\text{a}}_{15}\text{S}{\text{n}}_{4}\right) \end{array}$$

The charge and discharge curves are shown in Figure 6B. The charge and discharge tests were carried out at the current density of 50 mA g^−1^. In the initial discharge process, a short plateau appeared about 1.7 V, which corresponds to the formation of Na_x_SnS_2_ by sodiation into SnS_2_ layers. A tilted plateau from 0.8 to 0.6 V corresponds to the sodiation of more sodium ions into Na_x_SnS_2_, and the formation of metal Sn and Na_2_S. During this process, SEI film was formed at the same time (Luo et al., 2012). In the plateau below 0.6 V, a reaction occurred between metal Sn and sodium ions to form Na_x_Sn (in theory the x is less than 3.75). The discharge and charge capacities of the first cycle were 960.0 and 641.8 mAh g^−1^, respectively. The irreversible capacity loss of the first cycle was 318.2 mAh g^−1^, which is usually caused by the formation of SEI film. The charge and discharge curves of the second cycle were also shown in Figure 6B. The charge and discharge capacities in the second cycle were 645 and 677.1 mAh g^−1^, respectively. The charge curves in the first and second cycles almost overlapped, suggesting that the SnS_2_/rGO anode has a good electrochemical reversibility. The details of rate performance are shown in Figure 6C. SnS_2_/rGO electrode showed the discharge capacities of 645, 586, 536, 462, 387 and 320 mAh g^−1^ at 0.05, 0.1, 0.25, 0.5, 1.0 and 2.0 A g^−1^, respectively. The cycle performance was also investigated, as shown in Figure 6D. The electrode in the first four cycles was discharged and charged at 0.05 A g^−1^, and then at 0.25 A g^−1^. The capacity was 405 mAh g^−1^ after 100 cycles, and the capacity loss was 18.8%. Meanwhile, the coulombic efficiency was stable at about 99% for a long time after the first few cycles.

## Conclusion

In summary, SnS_2_/rGO nanosheet composite was synthesized by reflux condensation and hydrothermal methods. SnS_2_/rGO composite as an anode material showed excellent electrochemical properties for both LIBs and SIBs. The excellent sodium storage performance of the SnS_2_/rGO composite could be attributed to the following reasons. The nanosheet structure of SnS_2_ can shorten the diffusion path of lithium and sodium ions. Furthermore, rGO can enhance the electronic conductivity of the composite. Therefore, the SnS_2_/rGO composite could be considered as a promising anode material for LIBs and SIBs.

## Author Contributions

HC carried out the experiment and wrote the manuscript. HL, LM, DB, and SC participated in the experiment. BZ, JZ, WY, JZ, and ZD contributed to the discussion. HT supervised the experiment and proofread the manuscript.

### Conflict of Interest Statement

The authors declare that the research was conducted in the absence of any commercial or financial relationships that could be construed as a potential conflict of interest.
